# Fabrication of Si_3_N_4_@Si@Cu Thin Films by RF Sputtering as High Energy Anode Material for Li-Ion Batteries

**DOI:** 10.3390/ma14112824

**Published:** 2021-05-25

**Authors:** Hocine Merabet, Yannis De Luna, Khadiga Mohamed, Nasr Bensalah

**Affiliations:** 1 Department of Mathematics, Statistics and Physics, College of Arts and Sciences, Qatar University, Doha P.O. Box 2713, Qatar; km1306799@gmail.com; 2 Department of Chemistry and Earth Sciences, College of Arts and Sciences, Qatar University, Doha P.O. Box 2713, Qatar; yd1601559@student.qu.edu.qa

**Keywords:** Li-ion batteries, silicon-based anode, thin film, RF sputtering, performance

## Abstract

Silicon and silicon nitride (Si_3_N_4_) are some of the most appealing candidates as anode materials for LIBs (Li-ion battery) due to their favorable characteristics: low cost, abundance of Si, and high theoretical capacity. However, these materials have their own set of challenges that need to be addressed for practical applications. A thin film consisting of silicon nitride-coated silicon on a copper current collector (Si_3_N_4_@Si@Cu) has been prepared in this work via RF magnetron sputtering (Radio Frequency magnetron sputtering). The anode material was characterized before and after cycling to assess the difference in appearance and composition using XRD (X-ray Powder Diffraction), XPS (X-ray Photoelectron Spectroscopy), SEM/EDX (Scanning Electron Microscopy/ Energy Dispersive X-Ray Analysis), and TEM (Transmission Electron Microscopy). The effect of the silicon nitride coating on the electrochemical performance of the anode material for LIBs was evaluated against Si@Cu film. It has been found that the Si_3_N_4_@Si@Cu anode achieved a higher capacity retention (90%) compared to Si@Cu (20%) after 50 cycles in a half-cell versus Li^+^/Li, indicating a significant improvement in electrochemical performance. In a full cell, the Si_3_N_4_@Si@Cu anode achieved excellent efficiency and acceptable specific capacities, which can be enhanced with further research.

## 1. Introduction

Due to the continuous need for high-performance batteries, research interests in energy storage materials have become more significant as we move towards sustainable energy applications. Li-ion technology has remained at the forefront of energy research due to lithium’s high, variable discharge rate and specific capacity, as well as its small size [1] and relatively long cycle life, among others. The concept of Li-ion technology was originally proposed by Armand [2] as a method of improving the safety aspects of Li-based batteries through the use of intercalated electrodes. Today, the most commonly used Li-ion battery (LIB) involve intercalation materials, typically consisting of a cathode in the form of Li_x_M_y_O_2_ (M = Co, Ni, or Mn) [3,4] and a carbon material (i.e., graphite) [5] as the anode. Layered lithium metal oxides provide ≈170 mAh/g, whilst graphite has a low theoretical specific capacity of 372 mAh/g. This combination provides a specific energy of ≈150 Wh/kg [6], making it suitable enough for the small-scale applications it is found in today. However, large-scale applications, such as energy storage systems and electric vehicles, demand batteries with a much higher capacity and longer cycling capabilities [7].

Silicon as an anode material for LIBs presents a more attractive set of attributes that make it the best alternative to graphite. Aside from being the second most abundant element in the Earth’s crust, silicon is considered to be environment-friendly and cost-efficient [8,9], as opposed to lithium. Furthermore, Si offers a high specific capacity of about 4200 mAh/g based on the formation of Li_22_Si_4_ at high temperatures [8,10,11], which is over 10 times greater than that of graphite. This is due to the ability of silicon to form Li-rich alloys (i.e., Li_15_Si_4_, Li_21_Si_5_) with lithium, as opposite to graphite where six carbon atoms bond with only one Li^+^ ion (LiC_6_) [6,12]. In addition, silicon has a moderate working potential at 0.4 V vs. Li^+^/Li, making it a more viable candidate as an anode for LIBs with regards to energy density and safety aspects [13,14]. Despite these advantages, the use of silicon in commercial LIBs still proves to be unfeasible. The main obstacle to overcome with Si-based anodes is the large volume change of about 400% [8,15] that occurs with lithiation/delithiation processes. The constant expansion (lithiation) and contraction (delithiation) induce stress on the Si surface, resulting in cracks and active material pulverization [16]. The separation of the active material caused by the cracks results in a low electrical conductivity and prevents the transport of Li^+^ ions [17], which was initially provided by the addition of conductive carbon materials. Eventually, this leads to capacity fading and anode failure due to loss of electrical contact over long-term cycling [8]. At the same time, the alloying/dealloying of Li with Si leads to the formation of an unstable solid–electrolyte interface (SEI) on the anodic surface due to volume expansion/contraction [18]. In the first lithiation of a Si anode, SEI is formed and acts as a barrier between the anode and electrolyte, preventing further electrolyte decomposition. However, the SEI layer can breakdown after delithiation due to a reduced volume and reforms after repeated cycling, resulting in a thick layer and reduced anode performance [7].

The use of Si-based anodes in LIBs would have a wider reach than commercial graphite-based anodes, assuming that these challenges could be overcome. Recent developments on improving the performance of Si-based anodes include Si nanostructures (nanowires, hollow nanotubes) [19], core–shell structures (Si as core) [20,21], forming SiX alloys (X = Sn, Ge, etc.) [22,23], and surface coating, to name a few. In particular, coating the surface of Si with a conductive material, such as carbon [24] and copper [25], has been employed to improve the cycling performance of Si-based anodes in LIBs. However, such modifications still failed to surpass the performance of commercial LIBs. Some researchers have investigated the potential use of silicon nitride [26,27] and silicon nitride-based composites [28] as anode material for LIBs. The findings showed that these anode materials have a more stable cycling compared to silicon anodes. The downside to silicon nitride anode materials is that the specific capacities achieved are lower than theoretical values, with significant capacity fading after 100 cycles [26]. Researchers have speculated that the lack of elasticity in Si anodes contribute to the rapid capacity fading in LIBs [29]. Due to silicon nitride’s mechanical proprieties, its combination with Si as an anode material through nanostructuring and thin film coating may significantly reduce the volume change that accompanies alloying/dealloying processes. In this work, we have utilized thin film technology to improve the electrical conductivity of Si by sputtering a thin layer onto copper foil, along with the addition of silicon nitride (Si_3_N_4_) on top of the Si layer to accommodate the volume changes during lithiation/delithiation processes.

## 2. Experimental Section

### 2.1. Chemicals

Pure silicon and silicon nitride (Si_3_N_4_) were bought from Achemetal Tungsten & Molybdenum Co., Ltd (Henan, China). Lithium ribbon (thickness × W 1.5 mm × 100 mm, 99.9% trace metals basis) and organic carbonates (diethylene carbonate-DEC, dimethyl carbonate-DMC, and ethylene carbonate-EC) were obtained from Sigma-Aldrich (Saint Louis, MO, USA). Copper foil was purchased from MTI Corporation (Richmond, CA, USA).

### 2.2. Thin Film Deposition

Thin film deposition was carried out using a radio frequency (RF) magnetron sputtering system (PTL5S PVD, Plasma Technology Limited—PTL, Kowloon Tong, Hong Kong). Pure Si target was deposited on a copper (Cu) foil substrate (current collector) in a vacuum chamber for 6 h in the preparation of Si@Cu film. The same procedure was conducted for 1 h to achieve Si_3_N_4_@Cu film, with Si_3_N_4_ as the target. The combined Si_3_N_4_@Si@Cu film was involved the deposition of Si on Cu foil for 6 h, followed by Si_3_N_4_ on Si for 1 h. Each of the films were RF-sputtered with an RF power of 150 W. In order to achieve a uniform film deposition, substrates were rotated continuously with a stepper motor throughout the sputtering step. The process of sputtering was conducted at 5 × 10^−5^ Pa with a plasma pressure of 0.4 Pa in pure argon (working gas) at a flow rate of 10 sccm at 27 °C.

### 2.3. Characterization Techniques

X-ray diffractometry (XRD) (PAnalytical Empyrean X-ray diffractometer, 40 KV/30 mA, Malvern Panalytical Ltd., Cambridge, UK) was conducted at a scan rate of 2°/min between 20° and 90° to identify the crystal structures of each sample. Scanning electron microscopy (SEM) was carried out using an FEI Nova NanoSEM 450 (FEI Company, Hillsboro, OR, USA) to provide images of the deposited thin films, before and after cycling. Using the same instrument, energy dispersive X-ray (EDX) spectroscopy was completed to determine the composition of the elements in each sample. Transmission electron microscopy (TEM) was conducted using a FEI Tecnai G2 TEM TF20 (FEI Company, Hillsboro, OR, USA) to evaluate the films’ internal structures. X-ray photoelectron spectroscopy (XPS) was done using an AXIS Ultra DLD (Kratos Analytical Ltd., Manchester, UK) to identify the chemical distribution of elements on the films’ surfaces. Raman spectroscopy (Thermo Scientific™ DXR™ 2 Raman Microscope, Thermo Fisher Scientific, Darmstadt, Germany) was used to provide more information on the morphology of the as-prepared films. The thickness of the films was estimated using a Leica DCM8 Profilometer (Leica Microsystems, Wetzlar, Germany).

### 2.4. Coin Cell Preparation

The as-prepared films were cut into 16-mm disks using a precision disk cutter (MTI, MSK-T10, Richmond, CA, USA)Inside a glovebox (MTI, VGB-6-LD, Richmond, CA, USA), CR-2032 coin cells (MTI, VGB-6-LD, Richmond, CA, SUA) were assembled in a controlled atmosphere using an H_2_O and O_2_ purification system (<1 ppm of water and oxygen). The anode active material loading (in half cell and full cell) was 0.3 mg/cm^2^ Lithium metal disks were used as both counter and reference electrodes in half-cell configurations. In the full cell, LiFePO_4_ (LFP) was used as the cathode material. The LFP cathode was fabricated by mixing LiFePO_4_ powder with super P conductive carbon black (MTI, EQ-Lib-SuperP, Richmond, CA, USA), polyvinylidene fluoride (PVDF) in *N*-methyl-2-pyrrolidone (NMP) using a ball miller homogenizer for 1 h at 380 rpm. After ball milling, the obtained slurry was uniformly spread onto an aluminum foil (as the current collector) using a doctor blade (MTI, MSK-AFA-I, Richmond, CA, USA) to obtain a coating thickness of 5 µm. The composition of the slurry was 18/72/10 for C/LFP/PVDF, respectively. The cathode active material was directly cut into disks with a mass loading of 5 mg/cm^2^. A polypropylene membrane (Celgard, 2400, Asahi Kasei Corp., Tokyo, Japan) was used as the separator. A total of 20 μL of the electrolyte 1 M LiPF_6_ dissolved in EC/DMC/DEC (1:1:1 *v/v/v*) mixture was added.

### 2.5. Electrochemical Testing

Cyclic voltammetry (CV) tests were conducted using a CorrTest CS350 Potentiostat/Galvanostat Electrochemical Workstation (Wuhan Corrtest Instruments Corp., Ltd., Wuhan, China) at a scan rate of 0.01 mV/s. Galvanostatic charge/discharge (GCD) was carried out using an MTI 8 Channels Battery Analyzer BST8-WA (0.005–1 mA, up to 5 V, Richmond, CA, USA) at different current densities (20 and 1000 mA/g). The electrochemical performance of Si_3_N_4_@Si@Cu was evaluated in a full cell with lithium iron phosphate (LiFePO_4_; LFP).

## 3. Results and Discussion

### 3.1. Characterization of As-Prepared Anode Materials

**Figure 1:**
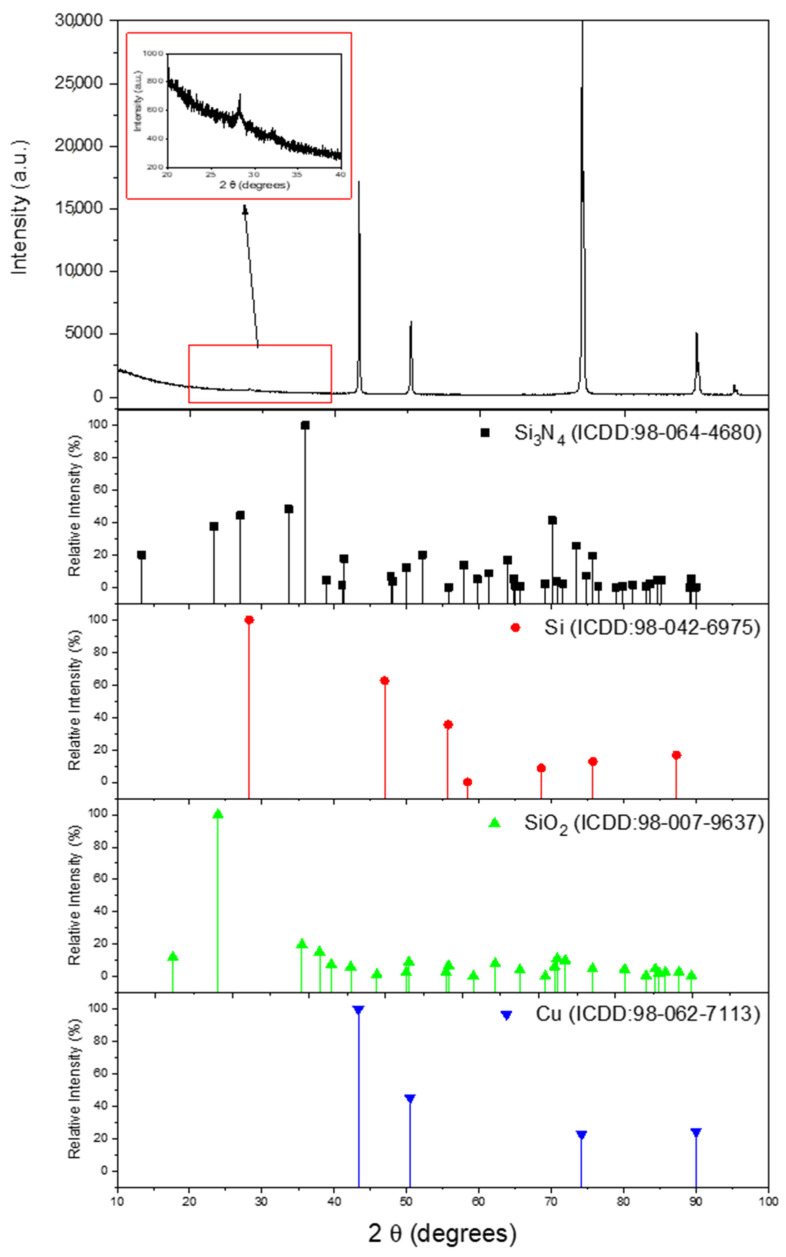
Composite XRD spectra of Si_3_N_4_@Si@Cu film in comparison with Si_3_N_4_, Si, SiO_2_, and Cu. The inset graph shows the relative intensity of Si_3_N_4_ in the sample.

Thin films were prepared (Si@Cu, Si_3_N_4_@Cu, and Si_3_N_4_@Si@Cu) using RF sputtering technique for various hours in a controlled environment (see Section 2.2). The as-prepared films were characterized using a range of analytical techniques: XRD, Raman spectroscopy, XPS, SEM, EDX, and TEM. The thicknesses of the films were evaluated after the sputtering process at specific times. The electrochemical performance of Si_3_N_4_@Si@Cu as the anode material in half cells was assessed against Li^+^/Li using electrochemical impedance spectroscopy (EIS), cyclic voltammetry (CV), and galvanostatic charge–discharge (GCD). The performance of the Si-based anode material was evaluated in a full cell with LFP. X-ray diffraction (XRD) analysis was conducted to confirm the deposition of Si and Si_3_N_4_ layers on the Cu sheet, as well as the detection of any compounds that might be present prior to cycling. The results from the XRD analysis are presented in Figure 1. From the spectra of the reference materials (Si_3_N_4_, Si, SiO_2_, and Cu), two peaks corresponding to Si_3_N_4_ were confirmed at 2θ = 71 and 90°. The majority of the peaks with the highest intensity coincided with the XRD spectrum of Cu. There were no peaks corresponding to Si and SiO_2_, indicating that the amorphous Si structure was deposited directly on the Cu substrate. This confirms the successful deposition of crystalline Si_3_N_4_ on a Si@Cu layer via RF magnetron sputtering.

**Figure 2:**
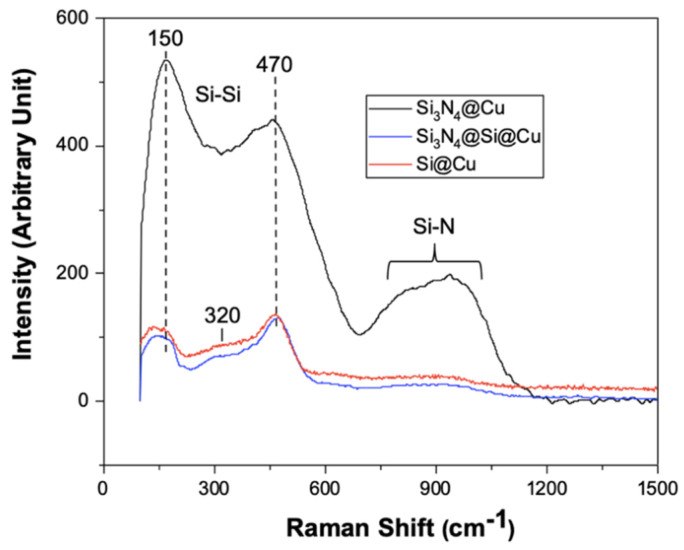
Raman spectra of Si-based anode materials deposited on a Cu sheet via RF magnetron sputtering.

Raman spectroscopy was carried out for the three Si-based thin films prepared using RF sputtering. The Raman spectra of the Si-based anode materials shown in Figure 2 give a clear distinction between Si_3_N_4_@Cu film and the other thin films, showing its much greater intensity. The spectra of Si@Cu and Si_3_N_4_@Si@Cu films are identical, with only a slight difference in intensity. The Raman spectrum for Si@Cu film (red) shows a peak at approximately 470 cm^−1^, as indicated in Figure 2, which occurs due to the Si–Si bond. According to studies on amorphous silicon [30,31,32], the characterizing peak of amorphous Si is a broad band centered at 480 cm^−1^, also known as the transverse optical (TO) peak. In addition, the absence of a sharp peak at 520 cm^−1^, typical of crystalline Si, confirms the formation of amorphous Si film in all as-prepared materials. The two smaller peaks, centered at approximately 150 and 320 cm^−1^, correspond to the transverse acoustical (TA) and longitudinal acoustical (LA) peaks of Si–Si bonds, respectively [32]. In silicon nitride films, Si–N bond vibrations appear broad and are typically between 700 and 1000 cm^−1^ [33,34]. These vibrations are evident in the Raman spectra of the Si_3_N_4_@Cu film.

**Figure 3:**
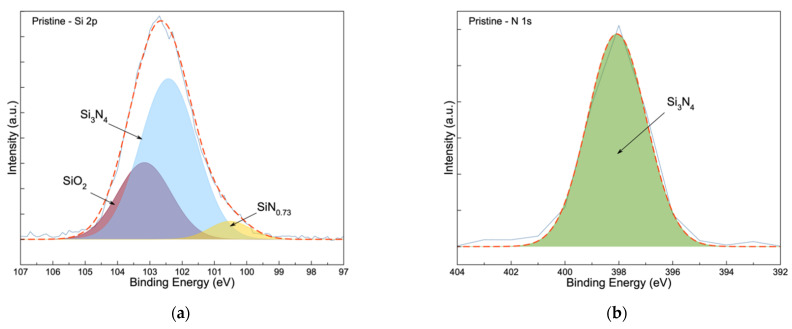

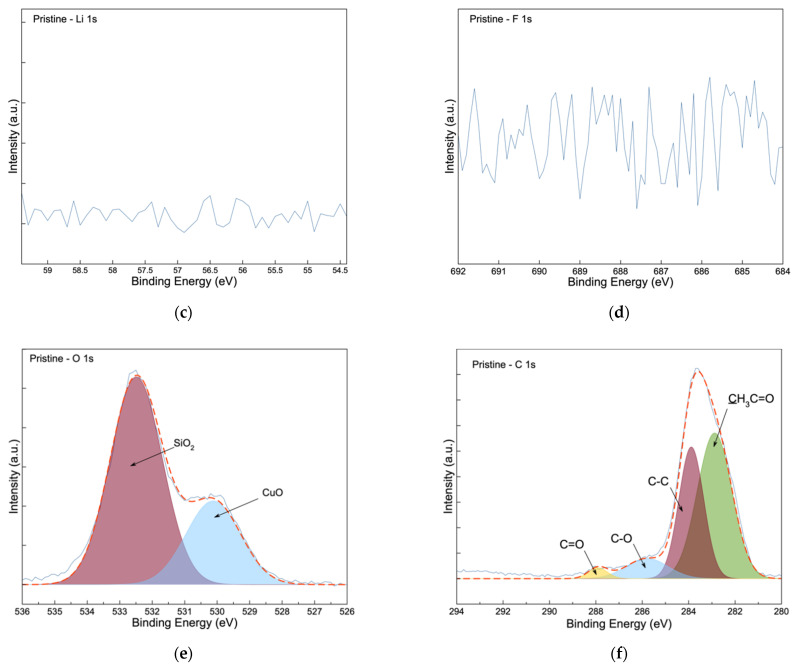
XPS spectra of (**a**) Si 2p, (**b**) N 1s, (**c**) Li 1s, (**d**) F 1s, (**e**) O 1s, and (**f**) C 1s of pristine Si_3_N_4_@Si@Cu anodes.

The pristine Si_3_N_4_@Si@Cu anode was subjected to X-ray photoelectron spectroscopy (XPS) analysis to determine the original composition of the anode’s surface prior to cycling. The XPS spectra of Si 2p, N 1s, Li 1s, F 1s, O 1s, and C 1s are given in Figure 3. The presence of oxygen could be due to the formation of oxides (CuO and SiO_2_) when the sample was exposed to air before and after the sputtering experiments. In the pristine anode, three peaks were observed in the Si 2p spectra (Figure 3a), which matched well with the binding energies of SiN_0.73_ (100.5 eV), Si_3_N_4_ (101.7 eV), and SiO_2_ (103.2 eV) reported in the literature [28,35]. These were further confirmed with the N1s and O1s spectra in Figure 3b,e, respectively. As expected, no peaks are observed in the Li 1s (Figure 3c) and F 1s spectra (Figure 3d) for the pristine Si_3_N_4_@Si@Cu anode, as no interaction occurred between the electrodes and electrolytes. Two peaks that are attributed to CuO and SiO_2_ were observed in the O 1s spectra, which occur naturally when exposed to the oxygen in air. The presence of four peaks in the C 1s spectra can be ascribed to the presence of residual acetone, which is used to clean the XPS chamber and the sample holder.

**Figure 4:**
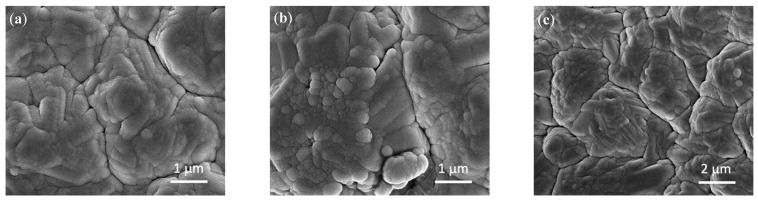
SEM images of (**a**) Si@Cu, (**b**) Si_3_N_4_@Cu, and (**c**) Si_3_N_4_@Si@Cu thin films prepared by RF sputtering.

SEM was carried out to produce images of the surface of each of the thin films prepared in the present work. Micrographs can help identify any differences between the films, as well as the effects of cycling on the Si_3_N_4_@Si@Cu anode material. Scanning electron micrographs are given in Figure 4. As shown in Figure 4a, an amorphous silicon structure formed on the surface of Cu foil due to the RF magnetron sputtering process, which was previously confirmed using XRD and Raman spectroscopy. Compared to the Si@Cu film, the surface of deposited Si_3_N_4_ (Figure 4b,c) appeared to be rougher with more spherical particles. The additional roughness on the surface and protective layer provided by Si_3_N_4_ may help in aiding with the volume expansion and contraction of the Si surface during battery cycling.

**Figure 5:**
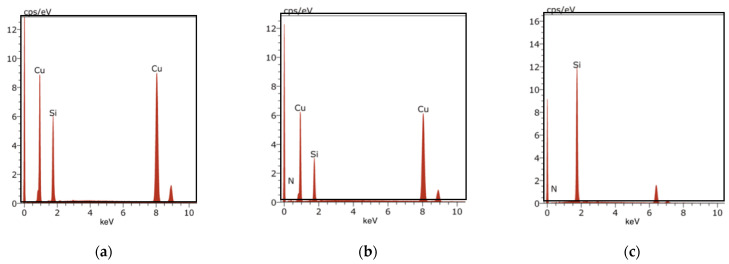
EDX analysis of (**a**) Si@Cu, (**b**) Si_3_N_4_@Cu, and (**c**) Si_3_N_4_@Si@Cu films.

Energy dispersive X-ray (EDX) spectroscopy was performed with SEM to identify the elemental composition of each prepared film. The results from the EDX analysis are shown in Figure 5. The analysis of the Si@Cu film confirmed the presence of Si and Cu, whilst the presence of N was also detected in the Si_3_N_4_@Cu film (the cps for nitrogen is 0.392 keV). As for the Si_3_N_4_@Si@Cu film, the presence of Si and N was confirmed. Based on these results, thin film deposition via RF magnetron sputtering generated the desired materials.

**Figure 6:**
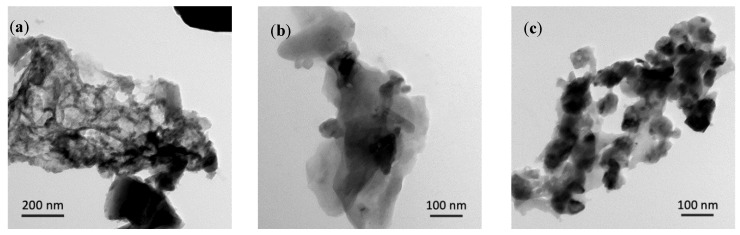
TEM images of (**a**) Si@Cu, (**b**) Si_3_N_4_@Cu, and (**c**) Si_3_N_4_@Si@Cu prepared by RF sputtering.

Transmission electron microscopy (TEM) was conducted to provide further information on the morphology of the as-prepared Si-based films beyond the surface. The first micrograph (Figure 6a) presents a network of amorphous silicon film, as opposed to the smooth structure in Figure 6b and the disconnected structure in Figure 6c. Evidently, the Si_3_N_4_@Si@Cu film has numerous spherical particles that are not present in the other Si-based films, as can be seen in Figure 6c. This micrograph shows a homogeneous layer of amorphous silicon with a silicon nitride layer.

**Figure 7:**
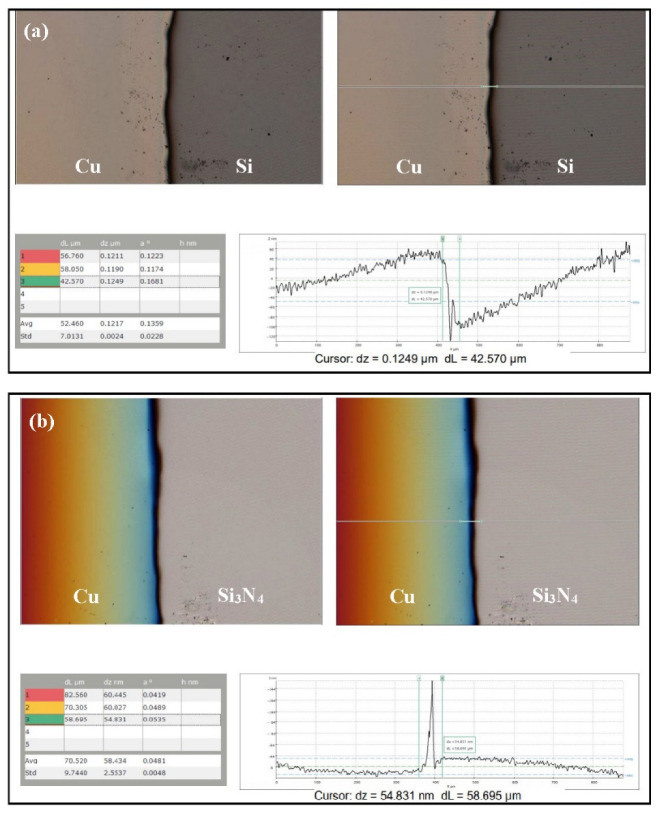
Measurements of film thickness of (**a**) Si@Cu and (**b**) Si_3_N_4_@Cu after 1-h deposition using RF magnetron sputtering.

Film thickness of the Si-based anode materials (Si and Si_3_N_4_) was measured by topography. The thickness of the Si film after a 1-h deposition time was estimated to be 124.9 nm, as shown in Figure 7a. Si was deposited on Cu using RF sputtering for 6 h, which gave an overall thickness of 749.4 nm. The approximate thickness of the Si_3_N_4_ film after a 1-h deposition was 54.8 nm (Figure 7b). Based on these values, the estimated thickness of the bilayer film (Si_3_N_4_@Si) was 804.2 nm.

### 3.2. Electrochemical Tests

**Figure 8:**
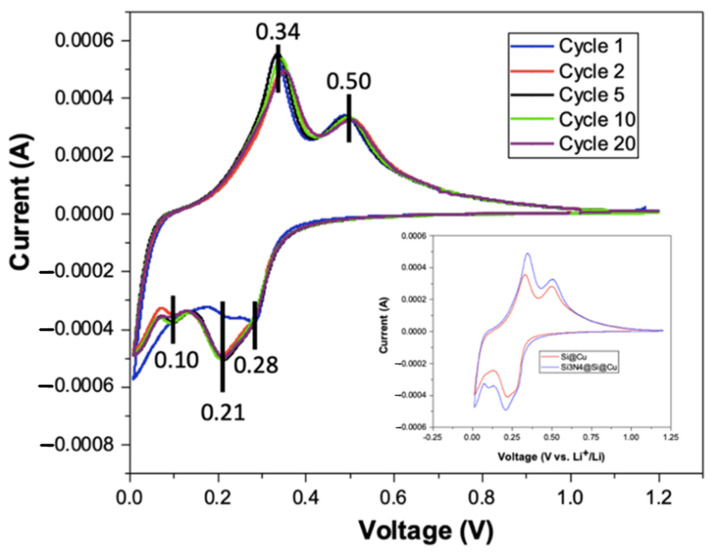
Cyclic voltammograms of Si_3_N_4_@Si@Cu anode in half cell vs. Li^+^/Li. Graphical inset features the first CV of Si_3_N_4_@Si@Cu compared with Si@Cu film.

The electrochemical performances of the Si-based films as anode material in half cells were assessed using various electrochemical tests. These included electrochemical impedance spectroscopy (EIS), cyclic voltammetry (CV), and galvanostatic charge–discharge (GCD). Cyclic voltammetry tests were conducted with Si_3_N_4_@Si@Cu as an anode in a half-cell using Li metal as a counter and reference electrodes for 20 cycles between 0.05 and 1.2 V vs. Li^+^/Li. As indicated in Figure 8, three peaks in the cathodic (reduction) region were identified, which correspond to the discharging (lithiation) of the battery. These peaks are located at 0.28, 0.21, and 0.10 V. In the anodic (oxidation) region, two peaks were identified, which are located approximately at 0.34 and 0.50 V. This corresponds to the charging of the battery in which delithiation occurs. Apart from one cathodic peak in cycle 1, the positions of each peak are identical and overlap with each other throughout the 20 cycles. This implies that the Si-based anode has excellent recyclability. In addition, the as-prepared anode material has shown good reversibility. In comparison with a previous literature on amorphous Si (conducted at 0.01 mV/s) [36], the voltammogram from this study is almost identical in terms of both the shapes and locations of peaks, except with one cathodic peak at 0.45 V that is only present in the CV plot of amorphous Si. On the contrary, CV curves for crystalline Si differ from the Si_3_N_4_@Si@Cu anode in this present work. The peaks for crystalline Si are less sharp and are shifted to lower voltages in the cathodic region [37]. Therefore, these observations further support the characterization findings that the Si_3_N_4_@Si@Cu anode has an amorphous Si instead of crystalline Si morphology. The inset in Figure 8 shows cycle 1 of the Si_3_N_4_@Si@Cu anode (blue) in comparison with Si@Cu anode (red). Both anode materials have two anodic peaks located at the same position in terms of voltage. However, three cathodic peaks were present for the Si_3_N_4_@Si@Cu anode, while only two peaks were observed for the Si@Cu film. Specifically, the peak located at 0.1 V is missing. Another difference is the intensity of the peaks, where the Si_3_N_4_@Si@Cu anode reached a higher intensity (current) than the Si@Cu anode.

**Figure 9:**
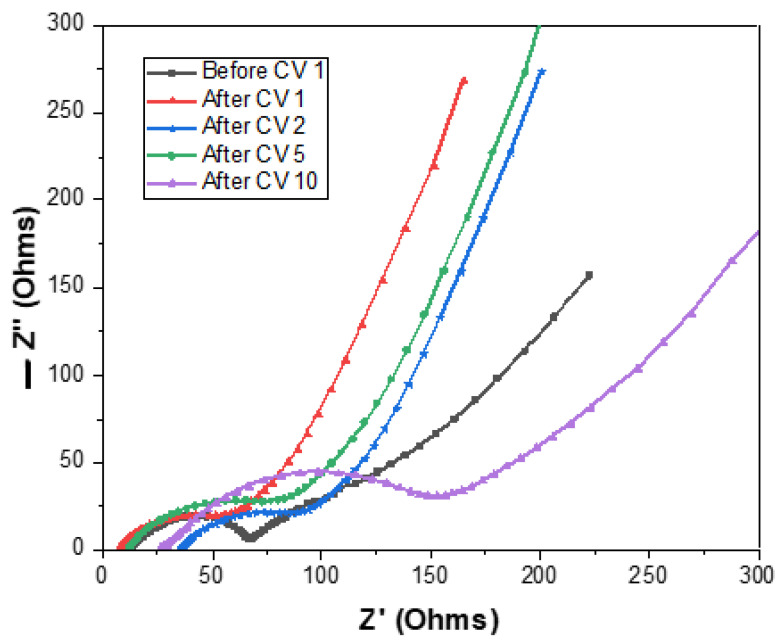
EIS Nyquist plot of Si_3_N_4_@Si@Cu anode in half-cell using Li metal as counter and reference electrodes.

The EIS Nyquist plots correlate to CV and are measured before the first CV run until after the tenth CV, as presented in Figure 9. The size of the arcs before and after CV 1 remain the same, whilst an increase in the arc height can be seen after CV 5 and CV 10. In addition, the Nyquist plot after CV 10 has the highest increase in arc height and resistance (Z’) due to a shift to the right. This follows the general trend in which resistance increases after numerous cycles due to conductivity.

**Figure 10:**
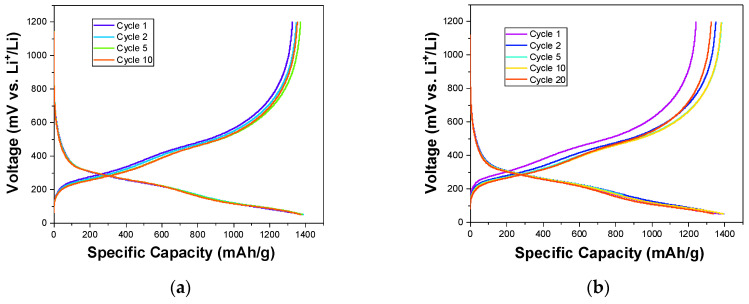

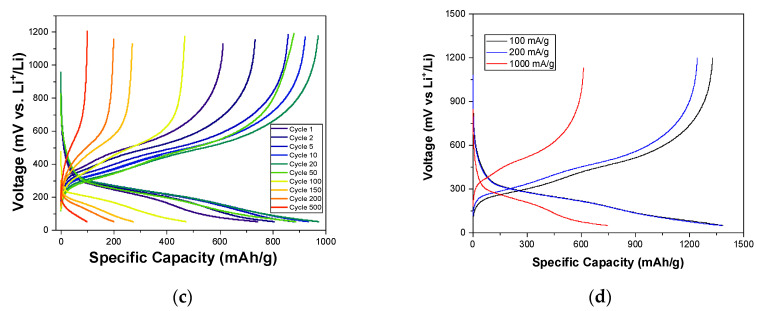
GCD profile of Si_3_N_4_@Si@Cu in a half cell at (**a**) 100 mA/g, (**b**) 200 mA/g, and (**c**) 1000 mA/g. (**d**) First GCD cycle of Si_3_N_4_@Si@Cu in a half cell at different current densities.

Galvanostatic charge–discharge (GCD) tests were performed to investigate the cycling behavior of Si_3_N_4_@Si@Cu as an anode material for LIBs when fully discharged and charged. The tests were carried out at various current densities to examine their effects on stability. The GCD plot carried out at 100 mA/g showed the highest stability and achieved the highest specific capacities in 10 cycles (Figure 10a). A similar plot is observed for a current density of 200 mA/g, but with a slightly lower stability during 20 cycles (Figure 10b). As the current density was increased to 1000 mA/g, the stability decreased throughout 500 cycles (Figure 10c). These observations are seen in the combined GCD plots of the first cycle at the three current densities in Figure 10d, wherein Si_3_N_4_@Si@Cu as an anode in a half cell performed significantly better at lower current densities.

**Figure 11:**
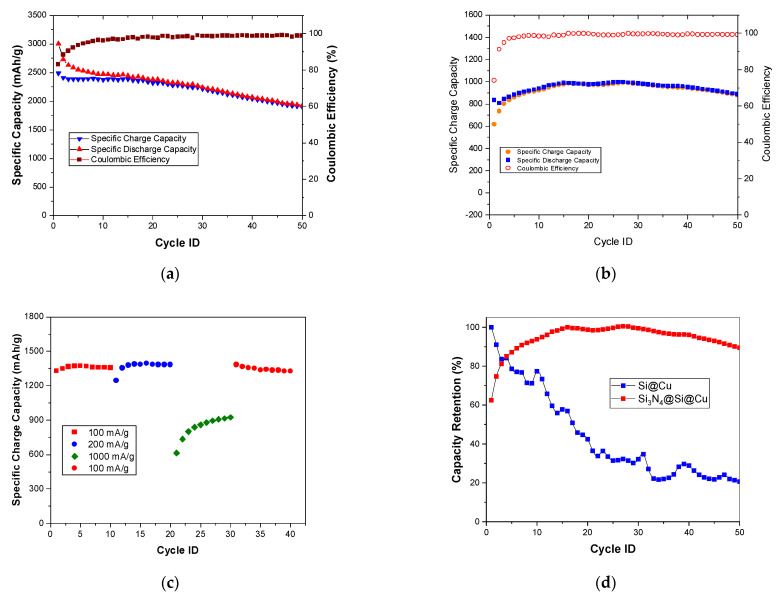
Cycling stability of Si_3_N_4_@Si@Cu in a half cell at (**a**) 20 mA/g and (**b**) 1000 mA/g. (**c**) Effect of capacity rate on performance (consecutive GCD). (**d**) Capacity retention of Si_3_N_4_@Si@Cu in comparison with Si@Cu.

Using the results from the GCD, the cycling stability of the Si_3_N_4_@Si@Cu anode was evaluated during 50 cycles at 20 and 1000 mA/g. The plots are presented in Figure 11. At a current density of 20 mA/g (Figure 11a), the discharge and charge capacities in the first cycle are 3000 and 2500 mAh/g, respectively. This led to an initial coulombic efficiency of 82%. After 15–20 cycles, the plots for specific charge and discharge capacities started to overlap, which is evident in the improved coulombic efficiency (99%). The specific capacity gradually decreased throughout the test until reaching 2000 mAh/g by the end of the 50th cycle. At a higher current density of 1000 mA/g (Figure 11b), lower discharge and charge capacities were attained in the first cycle (≈800 and 600 mAh/g, respectively). In the first 15 cycles, the specific capacity increased, followed by a slight decrease. This wave pattern was repeated twice then continued into a gradual decline in the last 10 cycles. However, the capacity values remained between 800 and 1000 mAh/g, suggesting a greater stability during cycling than at a lower current density. The coulombic efficiency achieved was initially 74%, which increased to 99% after about 15 cycles and then remained constant throughout cycling.

The charge capacity during consecutive GCD tests at increasing current densities (100, 200, and 1000 mA/g) was measured, and was then returned to 100 mA/g to investigate the effect of the capacity rate on performance (Figure 11c). In the first 10 cycles (100 mA/g), the charge capacity remained relatively constant at approximately 1350 mAh/g. The same trend was seen for a current density of 200 mA/g, with only a slight increase in charge capacity. Upon an increase in current density to 1000 mAh/g, the specific charge capacity dropped to 600 mAh/g and continued to increase until a maximum value just above 900 mAh/g was reached. When the current density was returned back to 100 mAh/g, the capacity gradually decreased from 1350 mAh/g. These results can be explained by a more severe structural evolution at lower rates due to in-depth repetitive Li-ion insertion and extraction compared to those at high rates. Furthermore, a remarkable difference in capacity retention was observed between Si_3_N_4_@Si@Cu and Si@Cu. The Si_3_N_4_@Si@Cu anode material achieved about 90%, whilst Si@Cu attained a 20% capacity retention. This indicates a significant battery performance for the as-prepared Si_3_N_4_@Si@Cu anode as opposed to Si@Cu film.

**Figure 12:**
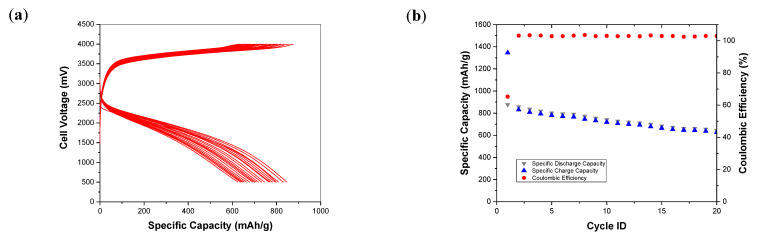
Electrochemical performance of Si_3_N_4_@Si@Cu//LFP full cell at 200 mA/g: (**a**) GCD profile and (**b**) cycling stability.

Following the half cell tests, the electrochemical performance of the Si_3_N_4_@Si@Cu anode was evaluated in a full cell combined with an LFP cathode at 200 mA/g, as presented in Figure 12. The GCD cycling of the full cell shows that the discharge and charge capacities were equal, initially below 900 mAh/g and then gradually decreased to 600 mAh/g. The coulombic efficiency of the cell was above 50% in the first cycle, which increased to 100% by the second cycle and remained constant throughout the 20 cycles. The battery delivered a satisfactory specific capacity and excellent efficiency; thus, a very good performance was observed from the anode material, which can be improved further in future studies.

### 3.3. Post-Mortem Analysis

**Figure 13:**
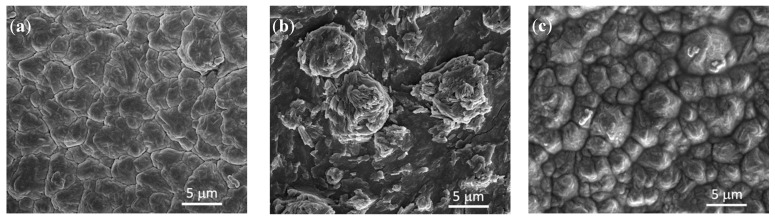
SEM images of Si_3_N_4_@Si@Cu anode material (**a**) before GCD, (**b**) after first discharge (lithiation) to 0.05 V vs. Li^+^/Li, and (**c**) after first GCD (lithiation/delithiation) to 1.20 V vs. Li^+^/Li.

Using SEM, the changes in the surface of the Si_3_N_4_@Si@Cu anode after lithiation and subsequent delithiation (first discharge to 0.05 V, first charge to 1.2 V) were examined and presented in Figure 13. Before the charge-discharge cycle, the micrograph of the as-prepared anode material displays a homogeneous layer of Si_3_N_4_. Upon the first discharge, also known as lithiation, the presence of elevated crystalline particles on the surface of the anode was observed (Figure 13b). This morphological change, which may imply an expansion in volume. After completing the first charge–discharge cycle wherein lithiation and delithiation occur, the surface of the anode material appears to return back to its original state, although slightly raised. This indicates that the volume change during lithiation/delithiation did not deteriorate the anode materials (no cracks were observed).

**Figure 14:**
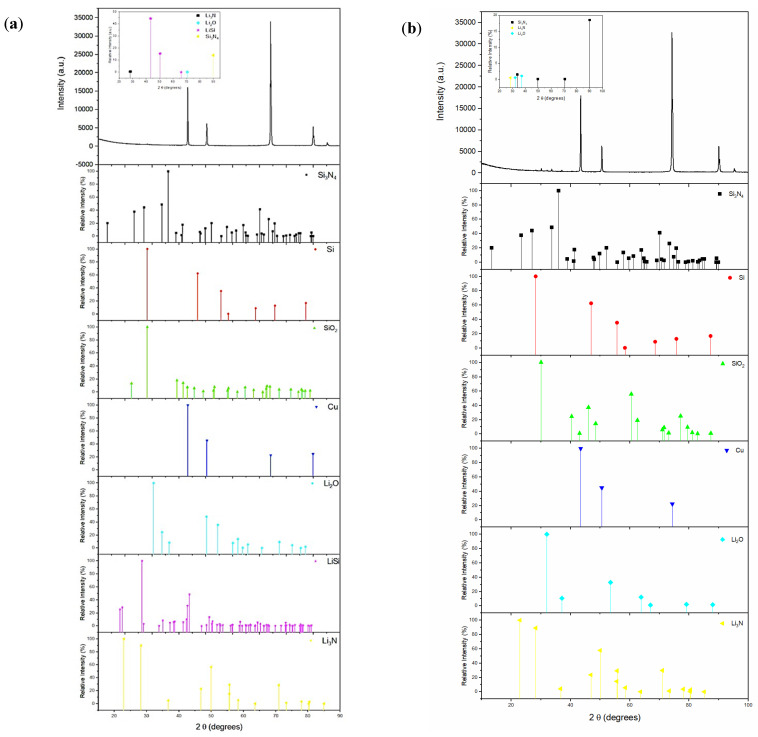
XRD spectra of Si_3_N_4_@Si@Cu anode material (**a**) after lithiation to 0.05 V and (**b**) after delithiation to 1.20 V in a half cell vs. Li^+^/Li. The standard XRD spectra of Si_3_N_4_ (ICDD:98-064-4680), Si (ICDD:98-042-6975), SiO_2_ (ICDD:98-005-1701), Cu (ICDD:98-062-7117), Li_2_O (ICDD:98-018-2028), LiSi (ICDD:98-008-3826), and Li_3_N(ICDD:98-003-478) are given for comparison.

XRD analysis was conducted after the charge and discharge processes to confirm whether the lithiation reaction has occurred and to identify the compounds that form after lithiation and delithiation. The results from the XRD analysis are presented in Figure 14. After lithiation (discharge), four compounds have been detected: Si_3_N_4_, Li_3_N, Li_2_O, and LiSi. The detection of Si_3_N_4_ confirms that this deposited layer remained on top of the Si layer to act as a protective barrier. The formation of LiSi, Li_3_N, and Li_2_O confirm the lithiation reaction, wherein Li^+^ ions react with Si, N, and O. This indicates the destruction of the anode following the reaction. After delithiation (Figure 14b), it is evident that LiSi is no longer present, which indicates a reversible reaction between Li and Si. Therefore, this confirms that the Si in the as-prepared anode is capable of undergoing a successful delithiation reaction during the charging process. However, the presence of Li_3_N and Li_2_O have been detected after delithiation, which may indicate that these compounds remained on the surface and became part of the SEI.

**Figure 15:**
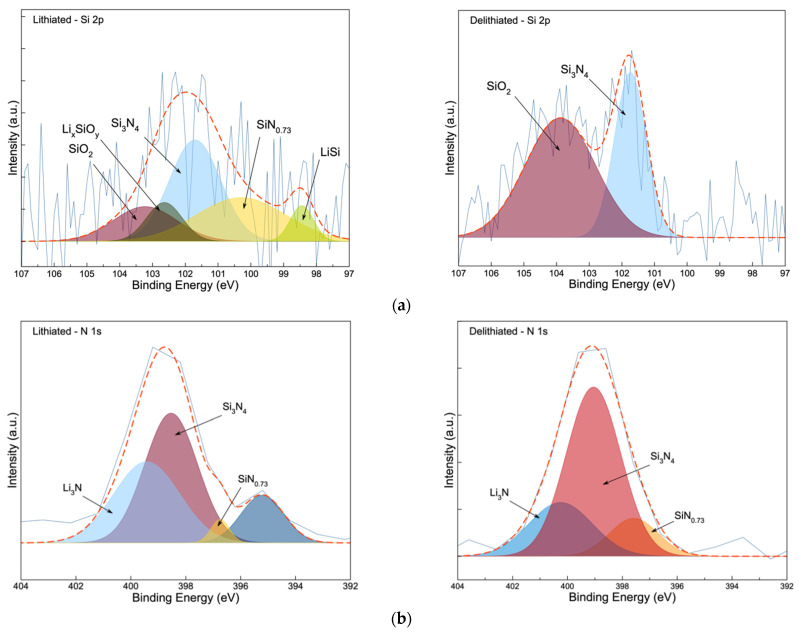

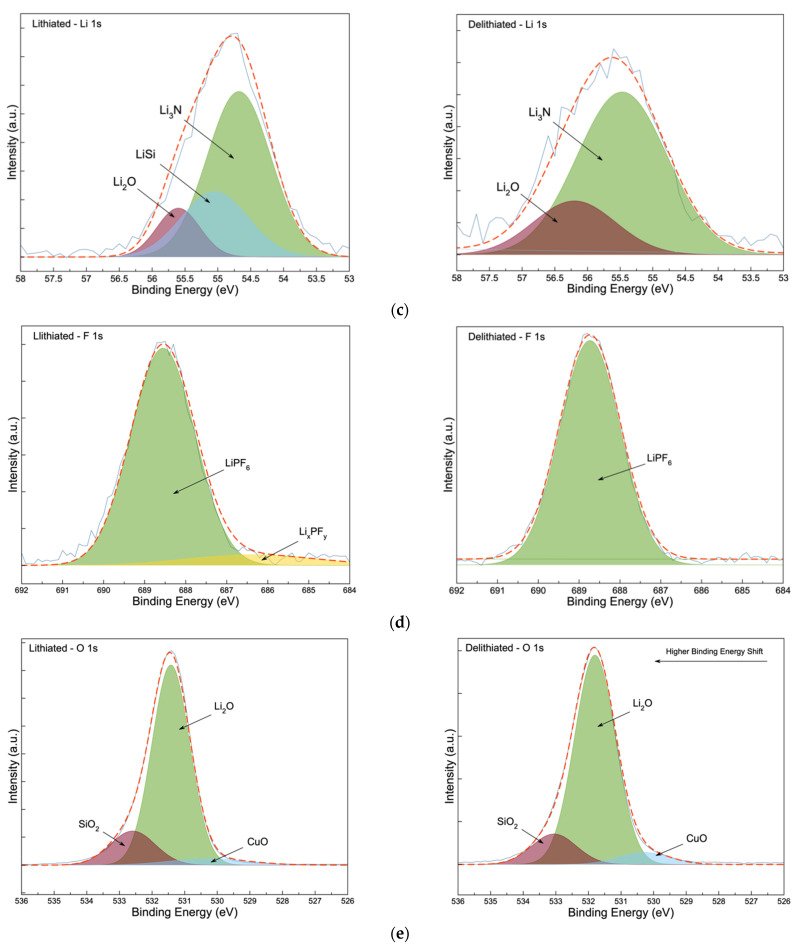

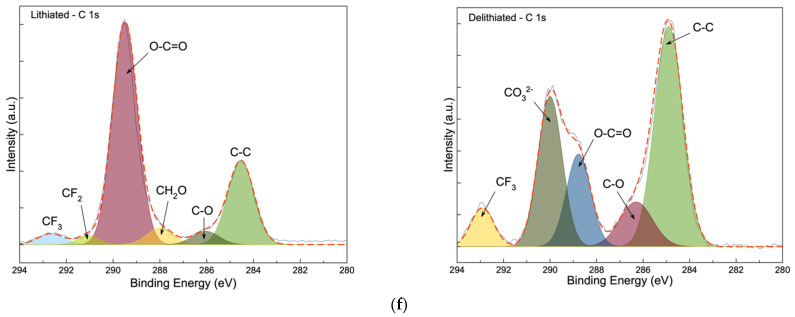
XPS spectra of (**a**) Si 2p, (**b**) N 1s, (**c**) Li 1s, (**d**) F 1s, (**e**) O 1s, and (**f**) C 1s at the surface of the Si_3_N_4_@Si@Cu anode during two stages: lithiated and delithiated (left to right).

To gain more understanding on the composition of the Si_3_N_4_@Si@Cu anode post-cycling, XPS was conducted after lithiation (0.05 V) and delithiation (1.20 V) in a half-cell vs. Li^+^/Li. Results from the XPS analysis are given in Figure 15. Five peaks were resolved in the lithiated Si 2p spectra (Figure 15a), three of which are the same compounds found in the pristine anode in Figure 3a (Section 3.1): SiN_0.73_, Si_3_N_4_, and SiO_2_ [28,35]. In addition to these three species, LiSi and Li_x_SiO_y_ were identified upon the lithiation of the anode, with peaks centered at 98.5 eV and 102.7 eV, respectively [38]. After delithiation, only Si_3_N_4_ and SiO_2_ were found to be present on the anode’s surface, which confirms the reversible lithiation/delithiation process. The presence of these Si-based compounds are also confirmed in the N 1s [28,35] and O 1s [39] spectra in Figure 15b,e, respectively. The main difference between the Li 1s spectra of the lithiated and delithiated anode is the existence of a peak at 55.1 eV in the lithiated stage, which is suspected to be LiSi that was observed in the XRD (Figure 14a). Otherwise, peaks that are attributed to Li_3_N and Li_2_O are present in both lithiated/delithiated phases, which were also confirmed in the N 1s and O 1s spectra [40]. The presence of the Li_3_N peak in the lithiated N 1s spectra and XRD analysis (Figure 14) confirms the involvement of the sputtered Si_3_N_4_ layer during lithiation. Two peaks were observed after deconvolution of the F 1s spectra of the lithiated anode in Figure 15d: LiPF_6_ (688.6 eV) (the supporting electrolyte) and Li_x_PF_y_ (686.2eV) [41]. In Figure 15e, the lithiated and delithiated anode have identical spectra with three peaks corresponding to CuO [42], Li_2_O [43], and SiO_2_ [39], with the exception of a higher binding energy shift observed in the delithiated phase. The lithiated and delithiated C 1s spectra in Figure 15f shows six and five peaks, respectively, that are attributed to various carbon bonds from the carbonates in the electrolyte, as well as the residual acetone that may have remained after cleaning the chamber and sample holder. Overall, XPS analysis has provided key insights and support for the results produced from the other analytical techniques, especially XRD analysis. The presence of Li_2_O, Li_3_N, and Li_x_PF_y_ in the lithiated and delithiated anode indicates the formation of a stable solid–electrolyte interface (SEI) by decomposition of the electrolyte. The stability of the SEI film confirms the good cycling stability of the Si_3_N_4_@Si@Cu anode.

## 4. Conclusions

In this endeavor, Si_3_N_4_@Si@Cu as an anode material for LIBs was prepared via RF magnetron sputtering. This work involves the deposition of Si_3_N_4_ on Si film as a possible solution to the cracking of the Si anode that results in poor electrochemical performance, which ultimately hinders the practical application of Si-based anodes in LIBs. Additionally, Si_3_N_4_@Cu and Si@Cu films were prepared for comparison. The pristine anode material was characterized using a series of analytical techniques: spectroscopy (Raman, XPS, EDX), microscopy (SEM and TEM), XRD analysis, and topography. Characterization results, mainly that of XRD and XPS, confirmed the successful deposition of Si_3_N_4_ on Si@Cu film through RF magnetron sputtering. Various electrochemical tests were conducted to evaluate its performance in a half-cell (vs. Li^+^/Li) and full cell, including CV and GCD. Upon cycling, the Si_3_N_4_@Si@Cu anode was found to perform better, with higher specific charge/discharge capacities, at lower current densities (100 and 200 mA/g). The most significant finding in this work was the superior performance of Si_3_N_4_@Si@Cu as LIB anode material compared to Si@Cu. The Si_3_N_4_@Si@Cu anode achieved 90% capacity retention after 50 cycles, whilst only 20% of the capacity was retained for Si@Cu. Furthermore, excellent efficiency (100% up to 20 cycles) and reasonably high specific capacities were attained for Si_3_N_4_@Si@Cu in a full cell with LFP, which already outperforms many commercial batteries today. Nevertheless, more work needs to be done in enhancing the specific capacity as the experimental value achieved in this work is still relatively low compared to the theoretical capacity. Post-mortem analysis was conducted to identify and assess any changes that occurred to the Si_3_N_4_@Si@Cu anode after cycling and during the lithiated/delithiated stages. The formation of Li_3_N was confirmed by XRD and XPS analysis in the lithiated and delithiated phases of the anode, which can be indicative of the role of Si_3_N_4_ as a protective barrier for Si.

## Data Availability

No new data were created or analyzed in this study. Data sharing is not applicable to this article.
